# SEM Study of Root Canal Walls Cleanliness after Ni-Ti Rotary and Hand Instrumentation

**Published:** 2007-04-01

**Authors:** Noosha Khadivi Nia Javan, Ladan Mohajeri Baradaran, Shahram Azimi

**Affiliations:** 1 *Dentist, private practice, Tehran, Iran*; 2 *Department of Endodontics, Dental school, Islamic Azad University of Medical Sciences, Tehran, Iran*

**Keywords:** Debris, K3, Race, Rotary Instrumentation, Smear Layer

## Abstract

**INTRODUCTION:** To compare the cleaning effectiveness of K3 and Race NiTi rotary systems with K-Flexo file instruments during the preparation of curved canals in extracted human teeth.

**MATERIALS AND METHODS:** A total of 50 root canals of mandibular and maxillary molars with curvatures ranging between 25º and 35 º were divided in three groups of 15 each and 5 as negative control groups. Canals were prepared using a low torque control motor in two rotary groups according to manufacturer’s instructions. Conventional Step back with S.S K files was the preparation technique in third group. The amount of debris and smear layer was quantified on the basis of a numerical evaluation scale. The data were statistically analyzed using Chi-Square and Likelihood Ratio tests.

**RESULTS:** In general, no significant difference in terms of amount of debris were found among three groups, only in apical zones of Race and K-Flexo file groups a significant difference was detected (P=0.041). Race rotary system left significantly less smear layer than k-Flexo file in the middle (P=0.009) and apical thirds (P=0.012), respectively. K3 significantly achieved higher scores of cleanliness than K-Flexo file in apical third only (P=0.049). No significant difference between Race and K3 rotary groups for residual debris and formed smear layer was detected.

**CONCLUSION:** Obtaining higher scores of cleanliness in various regions of the canals, crown down technique and the use of rotary instrumentation seem to be superior to conventional hand instrumentation with step back technique .No significant difference between K3 with three radial lands and Race with three cutting edges in terms of debris and smear layer formation was detected.

## INTRODUCTION

Successful root canal treatment depends, among other factors, on the removal of micro- organisms through chemo-mechanical instru-mentation of the root canal system. This includes the removal of the infected dentine and organic tissue by shaping and dissolution (1). All endodontic instruments create dentine debris and smear layer as a consequence of their action on root canal walls. This debris may be compacted along the entire surface of the canal walls, increasing the risk for bacteria contamination and reducing the adaptation of sealer and gutta-percha (2). Thus pulpal remnants, debris and smear layer must be totally removed (3). Some have suggested the protective role of smear layer against bacterial penetration into the underlying dentinal tubules (4). On the contrary, the presence of an infected smear layer may prevent antimicrobial agents from gaining access to the infected dentinal tubules. Furthermore, the removal of smear layer may enhance the adaptation of obturation materials to the root canal walls (3). Recently, rotary Ni-Ti root canal instruments have become an important part of the endodontic armamentarium (5). Advanced instrument designs including non-cutting tips, radial lands, different cross-sections and varying tapers have been developed to improve working safety, to shorten working time, and to create a greater flare of preparation (6). The design features of the cutting blade of endodontic instruments may affect the cleansing efficiency of the instruments (7). So the amount, thickness and the type of smear layer produced by new Ni-Ti instruments must be assessed (2).

Several studies (8-13,16) have shown that different rotary Ni-Ti instruments produced inconsistent results and that variations in their debris removal efficiency may result from variations in flute designs (4).

The ability of rotary instruments to remove dentine and pulpal debris during shaping is obviously connected to the flute and cross-sectional design (2,12). Wu and Wesselink (2001) determined that it may be difficult to instrument the entire wall in teeth with oval-shape canals and uninstrumented recesses may remain (14). Superior cleaning ability in the coronal and middle parts of the root canal has been described for various rotary Ni-Ti systems when compared to apical parts (1), partly because irrigants can only progress 1 mm further than the tip of the needle, and considering the dimensions of the dentine fragment/debris (greater than 15-20 µm) irrigant solutions may only partially contribute to their removal from the root canal space (2). According to some recent reports, instruments with sharp cutting edges seem to be superior to those having radial lands in cleaning of root canal (5,7).

The aim of this investigation was to compare the cleaning efficacy (residual debris and quality of the smear layer) after preparation of severely curved canals with Ni-Ti rotary Race (three cutting edges), K3 (three radial lands) versus conventional hand instrumentation with K-Flexo files.

## MATERIALS AND METHODS

A total of 50 extracted human maxillary and mandibular molars with intact crowns were selected. Coronal access was achieved using diamond burs and mesio-buccal canals were controlled for apical patency with a K-file size #10. Only teeth with intact apices were included. Standardized radiographs were taken prior to instrumentation with the initial file size #15 inserted in the mesio-buccal canals. Canals with a curvature between 25-35º, according to Schneider technique (8), were selected. The homogeneity of the three study groups (5 specimens each) and control group (5 specimens) with fair distribution of various degrees of curvatures and root lengths were visually observed.

The working length for all groups was obtained by measuring the length of the initial instrument (#10) at the apical foramen minus 1 mm. Each instrument was used to enlarge five canals only. After each instrument, the root canal was irrigated with 5mL of 2.5% NaOCl solution and at the end of instrumentation with 5mL of normal saline. The needle with a size 30 gauge (Supa, Tehran, Iran) was inserted as deep as possible into root canal without binding. For the two rotary groups , instruments were set in to 360º rotation with a 16:1 reduction contra-angle (W&H, Austria) power-ed by a torque-limited electric motor (ATR Tecnika, Milan, Italy). For each file, the individual torque limit and rotational speed were adjusted according to manufactures’ instructions. All procedures were performed by one operator. Control group cases were grossly irrigated with 10mL of 2.5% NaOCl solution only. In order to obtain similar final shapes EasyRace and VTVT packages of Race (Fkg Dentaire, La chaux-de-fonds, Switzerland) and K3 (SybronEndo, CA, USA) were used in a crown down approach. The size of master apical file in all cases was size #25. In manual instrumentation group, K-Flexo files (Maillefer Ballaigues, Switzerland) were used with step back technique, sizes #15 , 20, 25 were taken to full working length with a in and out movement and circumferential filing manner, then flaring using files #30, 35, 40 each size 1mm short of preceding instrument was completed.

After preparation, the specimens were stored in 100% relative humidity at 37ºC until further use. First the crowns and other roots were cut using a disc. A groove was prepared on the buccal and lingual surface of the tooth and split longitudinally with a mallet and chisel. Teeth showing evidence that the groove had penetrated into the root canal or exhibiting an irregular cleavage were discarded and replaced with a new specimen. Each halves were coded and mounted on an aluminum stab, coated with 200 Aº Gold-Palladium and examined in a scanning electron microscope (SEM) (Leo440i, Cambridge, England). Serial photomicrographs were taken at levels 2 and 6 mm below CEJ, and 2mm above apex.

**Figure 1:**
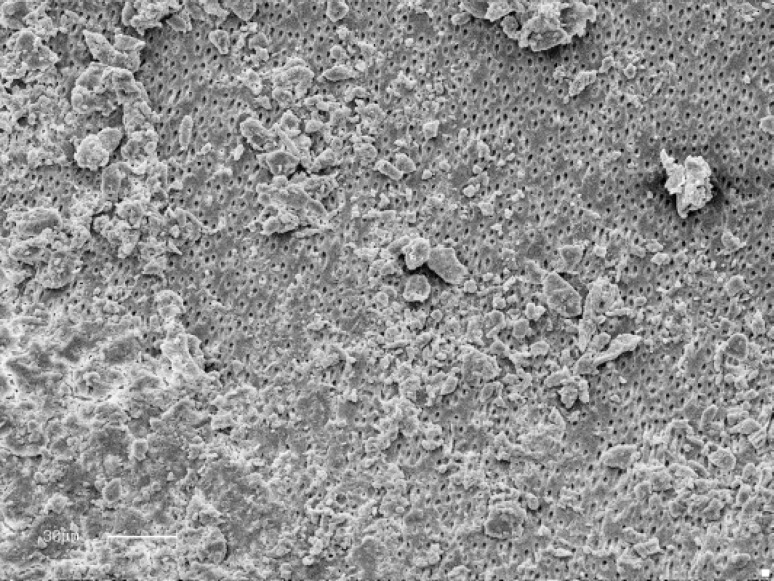
Un-instrumented canal irrigated with Sodium Hypochlorite 2.5%

Dentin chips, pulp remnants, large particles and aggregates appearing haphazardly on the root canal walls were classified as debris. A surface film consisting of remnants of dentine and pulp tissue with a smeared structure appearance was defined as smear layer. One hundred and fifty photomicrographs were coded and separately evaluated for debris and smear layer by means of a numerical evaluation scale (17) by three trained endodontists who were unaware of the procedure.

Numerical scores of specimens were converted to qualitative scales of “acceptable cleanliness” for scores 1 and 2; and “unacceptable cleanliness” for scores 3, 4, and 5 for both smear layer and debris scoring scales. The collected data were analyzed statistically with Chi-Square and Likelihood Ratio methods.

## RESULTS

At×900 magnification the instrumented canal walls in all groups appeared smooth and exhibited varying amounts of remaining debris and smear layer along the entire of the root canal (Figure 1). In some regions, grooves and un-instrumented area could be observed.

**Table 1:** Summary of scores of debris in three regions of the prepared canals

**Area** **System**	**Coronal** **(1,2) (3,4,5)**		**Middle** **(1,2) (3,4,5)**		**Apical** **(1,2) (3,4,5)**		***P*** ** value**
**K-flexo**	11^a^ (84.6)	2 (15.4)	9 (75)	3 (25)	5 (41.7)	7 (58.3)	0.059
**K3**	9 (69.2)	4 (30.8)	14 (93.3)	1 (6.7)	10 (71.4)	4 (28.6)	0.174
**Race**	10 (66.7)	5 (33.3)	14 (93.3)	1 (6.7)	13 (86.7)	2 (13.3)	0.142
***P*** ** value**	0.501		0.291		0.041		

***a: ***Number (Percent)

**Figure2:**
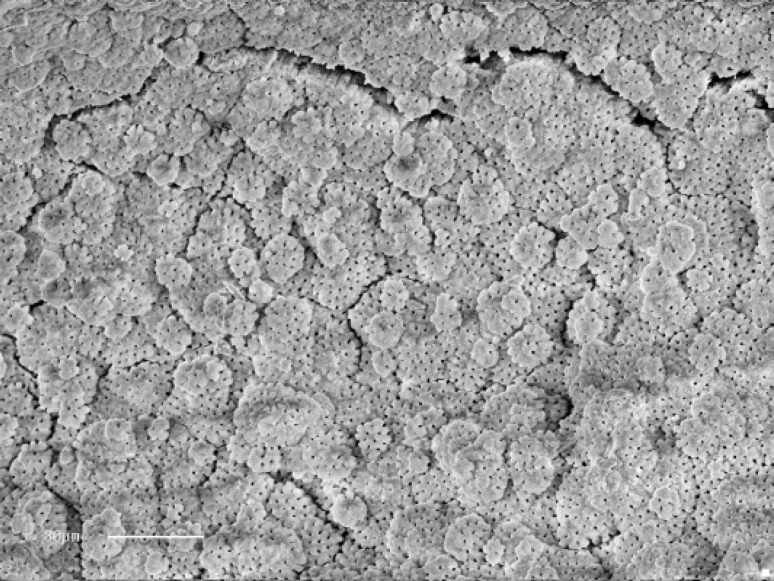
Smear layer and debris free area debris score: (1) smear layer: (1) (Original magnification 900)

Noticeably, the coronal and mid levels of all groups exhibited cleaner canal walls than apical levels. Completely clean root canals were not observed in any group. Typical globular appearance of uninstrumented dentine was observed in five specimens of control group (Figure 2). As expected no smear layer covering dentinal tubules, but pulp remnants and debris were detected in control group specimens. The mean scores of debris and smear layer recorded at three coronal, mid and apical levels are shown in table 1 and table 2. Recording the mean acceptable cleanliness scores for the entire areas as 67.6% for K-Flexo file, 78.6% for K3 and 82.2% for Race group, there were no statistically significant difference among the groups as to the elimination of debris in general. Comparing mean regional scores for debris residuals, there were no statistically significant differences in cleanliness of canal walls in coronal and mid levels of three groups.

**Table 2:** Summary of scores of smear layer in three regions of the prepared canals

**Area** **System**	**Coronal** **(1,2) (3,4,5)**		**Middle** **(1,2) (3,4,5) **		**Apical** **(1,2) (3,4,5)**		***P*** ** value**
**K-flexo**	2^a^ (15.4)	11 (84.6)	1 (8.3)	11 (91.7)	0 (0)	11 (100)	0.272
**K3**	5 (38.5)	8 (61.5)	4 (26.7)	11 (73.3)	3 (21.4)	11 (78.6)	0.611
**Race**	7 (46.7)	8 (53.3)	8 (53.3)	7 (46.7)	5 (33.3)	10 (66.7)	0.528
***P*** ** value**	0.18		0.031		0.041		

***a:*** Number (Percent)

**Figure 3:**
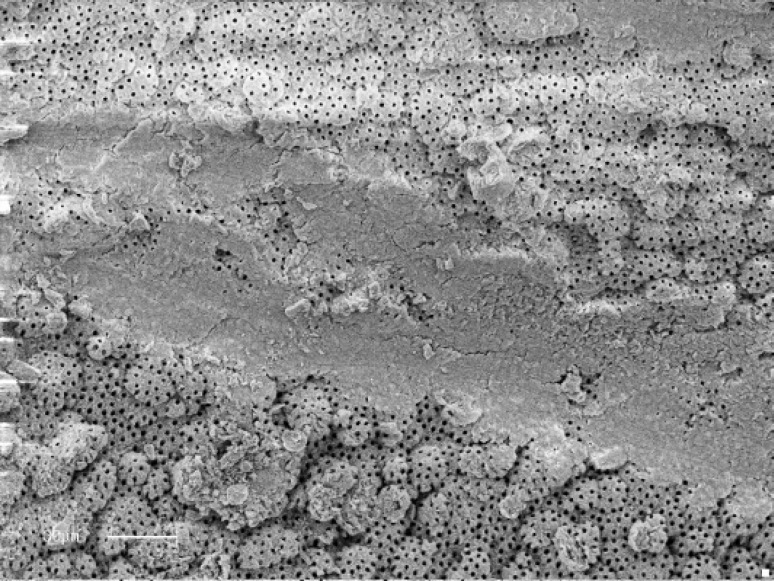
Less than 50% of canal wall covered with debris (Score 3) Some open dentinal tubules and partly smear layer covered region. (Score 2). (Original magnification ×900)

Acceptable cleanliness scores for debris in apical levels, 86.7% for Race, 71.4% for K3 and 41.7% for K-Flexo file group were recorded; while no statistically significant difference among two rotary groups was observed, there was a significant difference between Race and K-Flexo file groups in terms of debris amount in apical regions (Figure 3) (P=0.041).

Recording the acceptable cleanliness scores of smear layer for entire areas of root canals as 8.3% , 28.6% and 44.4% for K-Flexo file , K3 and Race groups respectively, while no statistically significant differences among the two rotary groups was detectable, both rotary groups significantly produced less smear layer than the K-flexo file in general (P<0.05).

In evaluation of smear layer scores at all levels of three groups, no significant differences in coronal areas of three groups were recorded. Noticeably, only 8.3% in mid and no accept-able cleanliness scores in apical regions of K-Flexo file group were recorded. Recording the 53.3% and 33.3% acceptable cleanliness scores for mid and apical levels of Race group respectively, there were a significant difference between Race and K-Flexo file groups in smear layer scores of mid and apical areas (P=0.009 and P=0.012).

**Figure 4:**
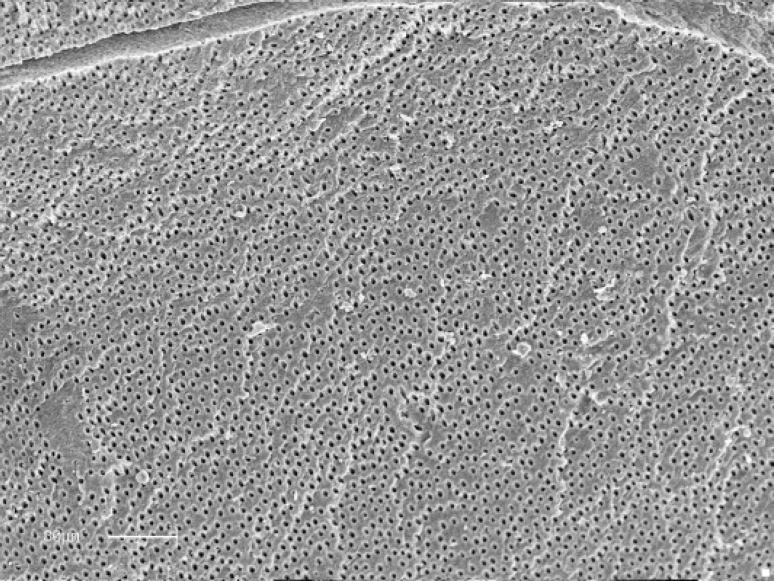
Path of file through un- instrumented globular dentin in apical zone (Original magnification ×nb 900)

Although higher acceptable cleanliness scores for smear layer in all regions of Race versus K3 group specimens were recorded, no statistically significant difference between the two rotary groups was detected. Interestingly, highest scores of canal cleanliness in terms of debris 93.3% and smear layer 53.3% in mid areas of Race group were recorded (Figure 4).

## DISCUSSION

SEM analysis appears to be an adequate method to investigate the influence of endodontic instruments on the morphology of dentine surfaces and has been well described (18). However, minor differences such as magnification, area selection, use of transparent grid assessment units and scoring systems might be noted.

In previous studies, different magnifications ranging from ×45 to ×2500 have been used (11, 15,19). At low magnification large amounts of debris can be easily seen, but details such as remnants of the smear layer or identification of dentinal tubules need to be observed at higher magnifications. A disadvantage of using higher magnification is the small size of the area of evaluation, potentially leading to misinterpret-tation. We found ×900 magnification to meet our study purposes.

Hülsmann *et al.* and Haikel and Allemann, (20, 21) proposed two separate five steps numerical evaluation scale for debris and smear layer scoring. Some others have used 3 or 4 step scoring systems. In our study, 3 endodontists were first familiarized with Shäfer and Schlingmann five step scoring system (17) and then the photomicrographs were provided for their evaluation.

In this study, the cleaning efficiency of the different instruments was assessed using two criteria, debris and smear layer. Debris was defined as dentine chips and residual vital or necrotic pulp tissue attached to root canal wall which in most cases is infected. The smear layer is a surface film of a thickness of approximately 1-2 µm. Smear layer, which is mainly inorganic, is performed when a canal is instrumented. As in our control group, no smear layer was detected in natural grooves and uninstrumented dentine surface specimens.

Although the influence of smear layer on outcome of the endodontic treatment is still controversial, it is considered to be desirable to remove the smear layer because of its potential deleterious effects (22). The effect of the combination of NaOCl and EDTA on the removal of smear layer, great portions of cir-cumferential dentine collagen and mineralized dentine wall from the most superficial part of tubules is well documented (18). Previous SEM studies investigated the effect of instrument-tation on dentine without EDTA (7,17,19,23) or with the use of EDTA gel (2,9,13) which the different methodologies used make a direct comparison of results difficult. Also a potential variable that may have affected the results is that the use of irrigants appeared to be less effective in areas that were not or partially instrumented (3). Considering the major object-tive of the present study, a simple irrigation technique was utilized. Thus, it should be noted that the cleaning efficiency of the three instruments evaluated in the present study might be further improved using a combination of NaOCl and EDTA.

In our study, with all instrumentation techni-ques, partially uninstrumented areas with remaining debris were found in all canal sections and on average the apical third of the canals were less clean than the middle and coronal thirds, regardless of the instrument used, which is in agreement with all previous studies. Peters *et al.* reported that approximately 35% of the canal surface area was not prepared when different Ni-Ti preparation techniques were used (4). In our study most uninstrumen-ted surfaces were found in coronal and mid levels of the two rotary groups which may be due to centering effect of Ni-Ti rotary files.

Selective instrumentation of canal walls may be the reason that uninstrumented areas were not detected in K-Flexo file group.

In Schäfer and Vlassis study, the use of Race instrument resulted in significantly less remaining debris compared to canal shaping with Protaper, whereas for smear layer no significant difference occurred (5), but cleanliness was not satisfactory for both systems in Paque *et al.* study (6). In Schäfer *et al.* study, the use of Mtwo instrument resulted in significantly less residual debris compared with canal shaping with K3 and Race instruments, whereas for smear layer no significant differences occurred (1).

Dentin chips cut by the Profile instrument underwent plastic deformation resulting in compressed 2-3 layers of smear with a shiny appearance and tree-bark configuration which may be the result of the heat generated along the cutting edge and burnishing action by radial lands (7).

Although not supported by our study results, there are some clues that the flute design of rotary Ni-Ti files may be a key factor for the cleaning efficiency of these instruments. Instruments with sharp cutting edges seem to be superior to those having radial lands (5,7).

## CONCLUSION

Both Race with triangular cross section-three cutting edges and K3 with three radial lands-three flutes cross section Ni-Ti rotary systems in a crown down manner proved to be superior in debris removal and smear layer formation over canal walls versus SS k-Flexo files with conventional hand instrumentation and step back technique.

## References

[1] Schäfer E, Erler M, Dammaschke T (2006). Comparative study on the shaping ability and cleaning efficiency of rotary Mtwo instruments. Part 2. Cleaning effectiveness and shaping ability in severely root canals of extracted teeth. Int Endod J.

[2] Foschi F, Nucci C, Montebugnoli L, Marchionni S, Breschi L, Malagnino VA, Prati C (2004). SEM evaluation of canal wall dentine following use of Mtwo and Protaper Ni-Ti rotary instruments. Int Endod J.

[3] Zmener O, Pameijer CH, Banegas G (2005). Effectiveness in cleaning oval-shaped canals using anatomic endodontic technology, Profile and manual instrumentation: a scanning electron microscopic study. Int Endod J.

[4] Pashley DH, Michelich V, Kehl T (1981). Dentin Permeability: effect of smear layer removal. J Prosthet Dent.

[5] Schäfer E, Vlassis M (2004). Vlassis M. Comparative investi-gation of two rotary nickel-titanium instruments: Protaper versus Race. Part 2. Cleaning effectiveness and shaping ability in severely root canals of extracted teeth. Int Endod J.

[6] Paque F, Musch U, Hülsmann M (2005). Comparison of root canal preparation using Race and Protaper rotary Ni-Ti instruments. Int Endod J.

[7] Jeon IS, Spångberg LS, Yoon TC, Kazemi RB, Kum KY (2003). Smear layer production by 3 rotary reamers with different cutting blade designs in straight root canals: A scanning electron microscopic study. Oral Surg Oral Med Oral Pathol.

[8] Hülsmann M, versümer J, Schade M (2000). A comparative study of Lightspeed, Profile 0.04, Quantec and Hero 642. Int Endod J.

[9] Hülsmann M, Schade M, Schäfers F (2001). A comparative study of root canal preparation with Hero 642 and Quantec SC rotary NI-TI instruments. Int Endod J.

[10] Hülsmann M, Gressmann G, Schäfers F (2003). A comparative study of root canal preparation using Flexmaster and Hero 642 rotary NI-TI instruments. Int Endod J.

[11] Schäfer E, Zapke K (2000). A comparative scanning electron microscope investigation of the efficacy of manual and automated instrumentation of root canals. J Endod.

[12] Gambarini G, Laszkiewicz J (2002). A scanning electron microscopic study of debris and smear layer remaining following use of GT rotary instruments. Int Endod J.

[13] Versümer J, Hülsmann M, Schäfers F (2002). Acomparative study of root canal preparation using Profile 0.04 and Lightspeed rotary NI-TI instruments. Int Endod J.

[14] Wu MK, Wesselink PR (2001). A primary observation on the preparation and obturation of oval canals. Int Endod J.

[15] Mayer BE, Peters OA, Barbakow F (2002). Effects of rotary instruments and ultrasonic irrigation on debris and smear layer scores: a scanning electron microscopic study. Int Endod J.

[16] Schnieder SW (1971). A comparison of canal preparation in straight and curved root canals. Oral Surg Oral Med Oral Pathol.

[17] Schäfer E, Schlingmann R (2003). Efficacy of rotary nickel-titanium K3 instruments compared with S.S hand K-Flexo file. Part 1: Cleaning effectiveness and instrumentation result in severely curved root canals of extracted teeth. Int Endod J.

[18] Torabinejad M, Hanysides R, Khademi AA, Bakland LK (2002). Clinical implication of the smear layer in endodontics: a review. Oral Surg Oral Med Oral Pathol.

[19] Ahlquist M, Henningsson O, Hultemby K, Olin J (2001). The effectiveness of manual and rotary techniques in the cleaning of the root canals: a scanning microscopy study. Int Endod J.

[20] Hülsmann M, Rümmelin C, Schäfers F (1997). Root canal cleanliness after preparation with different endodontic headpieces and hand instruments: a comparative SEM investigation. J Endod.

[21] Haikel Y, Allemann C (1988). Effectiveness of four methods for preparing root canals: a scanning electron microscopic evaluation. J Endod.

[22] Lim TS, Wee T, Choi MY, Koh WC, Sae-Lim V (2003). Light and scanning electron microscopic evaluation of Glyde File Prep in smear layer removal. Int Endod J.

[23] Schäfer E, Lohmann D (2002). Efficiency of rotary nickel-titanium Flexmaster instruments compared with stainless steel hand K-Flexofile. Part 2. Cleaning effectiveness and instrumentation results in severely curved root canals of extracted teeth. Int Endod J.

